# Connexins and gap junctions in the inner ear – it’s not just about K^+^ recycling

**DOI:** 10.1007/s00441-014-2029-z

**Published:** 2014-11-09

**Authors:** Daniel J. Jagger, Andrew Forge

**Affiliations:** UCL Ear Institute, University College London, 332 Gray’s Inn Road, London, WC1X 8EE UK

**Keywords:** Gap junction, Connexin, Inner ear, Cochlea, Deafness, Vestibular

## Abstract

Normal development, function and repair of the sensory epithelia in the inner ear are all dependent on gap junctional intercellular communication. Mutations in the connexin genes *GJB2* and *GJB6* (encoding CX26 and CX30) result in syndromic and non-syndromic deafness via various mechanisms. Clinical vestibular defects, however, are harder to connect with connexin dysfunction. Cx26 and Cx30 proteins are widely expressed in the epithelial and connective tissues of the cochlea, where they may form homomeric or heteromeric gap junction channels in a cell-specific and spatiotemporally complex fashion. Despite the study of mutant channels and animal models for both recessive and dominant autosomal deafness, it is still unclear why gap junctions are essential for auditory function, and why Cx26 and Cx30 do not compensate for each other in vivo. Cx26 appears to be essential for normal development of the auditory sensory epithelium, but may be dispensable during normal hearing. Cx30 appears to be essential for normal repair following sensory cell loss. The specific modes of intercellular signalling mediated by inner ear gap junction channels remain undetermined, but they are hypothesised to play essential roles in the maintenance of ionic and metabolic homeostasis in the inner ear. Recent studies have highlighted involvement of gap junctions in the transfer of essential second messengers between the non-sensory cells, and have proposed roles for hemichannels in normal hearing. Here, we summarise the current knowledge about the molecular and functional properties of inner ear gap junctions, and about tissue pathologies associated with connexin mutations.

## Introduction

**Fig. 1:**
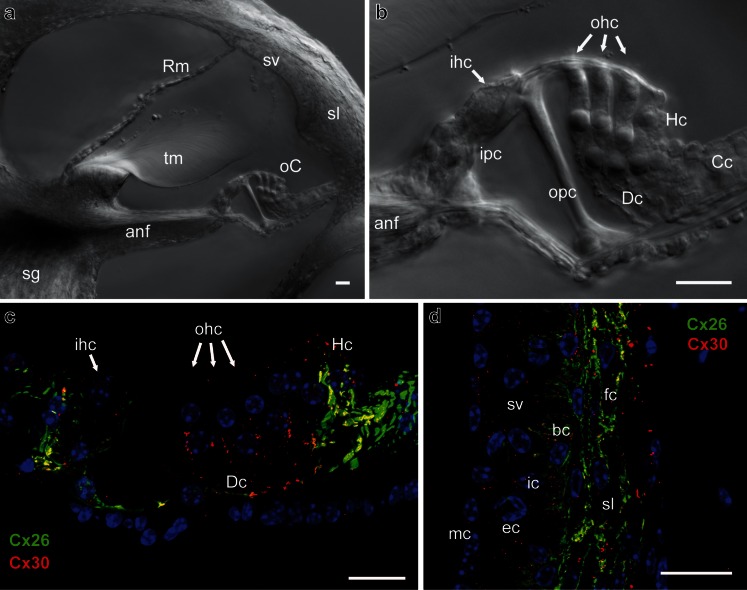
Expression patterns of Cx26 and Cx30 in epithelial and connective tissues of the cochlea. **a** A section of the cochlear apical turn of a P30 mouse showing the key sensory and non-sensory tissues involved in auditory sensory transduction. **b** Higher magnification of the organ of Corti showing the inner hair cells and outer hair cells separated by the tunnel of Corti, formed by the inner pillar cells and outer pillar cells. Adjacent to the outer hair cells are the Deiters’ cells, Hensen’s cells and Claudius’ cells. Immunofluorescent labelling of the organ of Corti (**c**) for Cx26 (*green*) and Cx30 (*red*) reveals a differential pattern of the connexins. Nuclei are stained using DAPI. Within the Deiters’ cell region, there are mostly Cx30-labelled gap junction plaques, whereas double-labelled plaques are evident elsewhere. In the connective tissue region of the cochlear lateral wall (**d**), most gap junction plaques are double-labelled for Cx26 and Cx30. *anf* auditory nerve fibres, *bc* basal cell, *Cc* Claudius’ cell, *Dc* Deiters’ cell, *ec* endothelial cell, *fc* fibrocyte, *Hc* Hensen’s cell, *ic* intermediate cell, *ihc* inner hair cell, *ipc* inner pillar cell, *mc* marginal cell, *oC* organ of Corti, *ohc* outer hair cell, *opc* outer pillar cell, *Rm* Reissner’s membrane, *sg* spiral ganglion, *sl* spiral ligament, *sv* stria vascularis, *sm* scala media, *tm* tectorial membrane. *Scale bars* 20 μm

The vertebrate inner ear consists of the hearing organ, the cochlea, and the vestibular system that detects changes in head position thereby contributing to maintenance of balance. Inner ear function is dependent on tightly controlled ionic environments, in particular for potassium ions (K^+^), which are the main charge carrier for sensory transduction. The inner ear contains two major fluid spaces, which are separated from each other by tight junction barriers. Scala media in the cochlea (Fig. 1a) and the vestibular semi-circular canals are filled with endolymph, which is rich in K^+^ but poor in Na^+^, thus more resembling an intracellular fluid. The remaining extracellular spaces within the inner ear contain perilymph, which has a high Na^+^ and low K^+^ concentration. The essential driving force for sensory transduction in the mammalian cochlea (but not in the vestibular system) is provided by the high positive potential of the endolymph (ca. 80 mV), the endocochlear potential (EP) (Wangemann 2006). In the cochlea, the ionic composition of the endolymph and the EP are generated and maintained by the stria vascularis, a stratified ion-transporting tissue that lines the lateral wall of the cochlear duct. The sensory epithelia contain the mechano-sensory receptors, known as hair cells, each of which is surrounded by non-sensory supporting cells (Fig. 1b). Supporting cells throughout all vertebrate classes are extensively coupled by large (>10-μm^2^) gap junctions (Forge et al. 2003a,b), some of which are amongst the largest in the body containing several hundred thousand connexon channels. The interconnection of most cells within the inner ear via gap junctions is thought to play a role in fluid homeostasis and/or intercellular signalling (Kikuchi et al. 2000a; Zhao et al. 2006).

## Gap junctions in the inner Ear

Based on ultra-structural studies, two gap junction networks—the gap junction system of the epithelial tissue and that of the connective tissue—have been described within the mammalian cochlear duct (Kikuchi et al. 1995) and the vestibular system (Kikuchi et al. 1994). The epithelial gap junction system connects the supporting cells of the sensory epithelia and bordering epithelial cells. In the cochlea, gap junctions are present between the supporting cells of the organ of Corti (Fig. 1c) and the root cells whose processes penetrate the spiral ligament (Jagger and Forge 2013). No gap junction plaques have been identified between hair cells and supporting cells. The syncytial nature of supporting cells and the segregation of hair cells from this functional unit has been confirmed by electrophysiology (Santos-Sacchi and Dallos 1983; Oesterle and Dallos 1990) and dye tracer studies in cochlear slices (Jagger and Forge 2006; Taylor et al. 2012; Forge et al. 2013). The gap junction system of inner ear connective tissues comprises the cells of the ion-transporting epithelium, various types of fibrocytes, and mesenchymal cells that line the scala vestibuli. In the cochlear duct, gap junctions are present between the fibrocytes of the spiral limbus and of the spiral ligament (Fig. 1d). Numerous gap junctions have been found between adjacent basal cells of the stria vascularis, between basal cells and intermediate cells, and between basal cells and fibrocytes of the spiral ligament. No gap junctions have been identified between the intermediate and the marginal cells of the stria vascularis, nor between adjacent marginal cells (Kikuchi et al. 1995; Forge et al. 2003b), suggesting that these cells are isolated from a functional unit formed by intermediate cells, basal cells and fibrocytes of the spiral ligament. Dye-coupling experiments in cochlear slices have confirmed the specific connectivity of spiral ligament fibrocytes with basal/intermediate cells, and the functional isolation of this unit from the marginal cells (Kelly et al. 2011).

## Connexin expression in the inner ear

The predominant connexins in the mammalian inner ear are Cx26 and Cx30 (see Fig. 1c, d), which are present in cells comprising the epithelial and connective tissue gap junction systems of the cochlea and vestibule (Kikuchi et al. 1994, 1995; Lautermann et al. 1998; Ahmad et al. 2003; Forge et al. 2003b; Sun et al. 2005; Jagger and Forge 2006; Liu et al. 2009). Furthermore, double immunofluorescence revealed overlapping labelling patterns for Cx26 and Cx30, suggesting a co-localisation of the two connexin isoforms within some gap junction plaques in the inner ear (Lautermann et al. 1998; Forge et al. 2003c; Sun et al. 2005; Liu et al. 2009). This has been supported by co-immunoprecipitation of Cx26 and Cx30 (Ahmad et al. 2003; Forge et al. 2003b) and by immuno-gold labelling of thin sections, where Cx26 and Cx30 were evenly distributed within the gap junction plaques (Forge et al. 2003b).

Cx31 is apparently confined to the spiral limbus and a particular population of fibrocytes in the spiral ligament and supra-strial zone, where it gradually decreases from base to apex (Xia et al. 2000; Lopez-Bigas et al. 2002; Forge et al. 2003b). In the rat cochlea, weak immunostaining of Cx43 has been reported between supporting cells in the organ of Corti, the stria vascularis and spiral limbus (Suzuki et al. 2003). This is contrary to the postnatal mouse, where Cx43 is confined to type III fibrocytes lining the inside of the bony wall, and the bone of the otic capsule, with no expression in the organ of Corti (Forge et al. 2003b; Cohen-Salmon et al. 2004). In the spiral ligament of the adult guinea pig, Cx43 immunofluorescence has been localised within the type III fibrocytes, specifically to intercellular plaques and intracellular structures consistent with a classical trafficking pathway (Kelly et al. 2012). Cx29 is expressed in the Schwann cells surrounding the auditory and vestibular myelinated fibres of ganglion neurons (Eiberger et al. 2006; Tang et al. 2006). In the avian inner ear, the chicken orthologs to the mammalian Cx30, Cx26, and Cx43 have been identified as major connexin isoforms (Nickel et al. 2006). Interestingly, chicken Cx30 is exclusively expressed in inner ear tissues and is present in all cells comprising the gap junction networks of the sensory and ion transporting epithelia of the cochlear duct and utricle (Heller et al. 1998; Nickel et al. 2006).

## Inner ear gap junctions and K^+^ homeostasis

The serial arrangement of gap junction networks within two distinct cochlear tissues has prompted the theory that gap junctions form the structural pathways for K^+^ re-circulation within cochlear and vestibular tissues (Kikuchi et al. 2000b; Wangemann 2002; Jagger and Forge 2013). In this model, K^+^ ions exiting hair cells during auditory transduction are siphoned from the extracellular perilymph by supporting cells immediately adjacent to hair cells. The ions are then relayed radially via the epithelial gap junction network to the connective tissue gap junction network, where they may be taken up by fibrocytes of the spiral ligament or the vestibular connective tissue and secreted back into the endolymph by the marginal cells of the stria vascularis or the vestibular dark cells. This model is derived largely from indirect evidence, often in the form of protein expression data, and still requires a substantial amount of support from direct physiological evidence. The importance of buffering K^+^ in perilymph is clear, however; depolarisation-induced damage to hair cells and neurons would lead to permanent hearing impairment.

A homeostatic model can be extended from the “spatial K^+^ buffering” observed in glial cell networks in the brain (Kofuji and Newman 2004). Here, the extensive cell syncytium acts as a K^+^ sink, allowing redistribution of the K^+^ flux to regions of inactivity. The extensive connexin expression in the epithelial and connective tissue networks along the cochlear partition reveals a substantial buffering volume. The K^+^-buffering theories have a number of potential problems, not least the inability of individual connexins (either Cx26 or Cx30) to compensate for the loss of the other (Cohen-Salmon et al. 2002; Teubner et al. 2003). Both Cx26 and Cx30 homotypic channels can transfer K^+^ ions (Valiunas et al. 1999; Manthey et al. 2001), and so should ably manage K^+^ when expressed alone in the cochlea. Acceptance of the K^+^ recirculation model has been limited by the lack of evidence of K^+^ transfer from the epithelial gap junction network to the connective tissue gap junction network. However, our recent work demonstrated the existence of weakly rectifying K^+^ currents through the basolateral processes of root cells, which were likely mediated via Kir4.1 channels (Jagger et al. 2010). Furthermore, the Kir4.1 channels are co-localized with aquaporin channels (Eckhard et al. 2012), suggesting that large K^+^ fluxes could occur without disturbance of the hydrostatic microenvironment of the spiral ligament.

## The biophysical characteristics of cochlear gap junctions

The properties of cochlear gap junctions have been determined in native cochlear tissue, using combinations of in vivo and ex vivo preparations, and in transfected cell lines expressing Cx26 and/or Cx30. Homotypic Cx26 or homotypic Cx30 channels have been studied extensively in cell culture systems using the double patch clamp technique (Dahl et al. 1996; Valiunas et al. 1999; Beltramello et al. 2003, 2005). Considering the high sequence homology between Cx26 and Cx30 (Dahl et al. 1996), the properties of these channel types are surprisingly distinct, particularly in terms of conductance and voltage gating. Unfortunately, there are few, if any, patch clamp data from cell lines co-transfected with Cx26 and Cx30. However, the voltage-dependence and gating properties of gap junction channels in small groups of supporting cells have been determined. The voltage-dependence of gap junctions in Hensen’s cells could be grouped into four distinct types (Zhao 2000). These gating responses showed varying degrees of polarity-dependent or polarity-independent rectification. The observation of extensive asymmetric voltage gating points to a complexity in gap junctional coupling which argues in favour of channels composed of non-homotypic connexin types. In single recordings, it is possible that multiple channel types contribute to the observed complex responses. Channels between adjacent Deiters’ cells may also allow rectification of current flow (Zhao 2000). There is evidence from patch clamp recordings that the gap junctional conductance between cochlear supporting cells can be modulated by intrinsic factors such as nitric oxide (Blasits et al. 2000) or turgor pressure (Zhao and Santos-Sacchi 1998).

## The molecular selectivity of cochlear gap junctions

The molecular selectivity of cochlear gap junctions may be of equal importance to their electrophysiological properties. There is increasing evidence that intercellular fluxes of second messengers such as inositol phosphates and Ca^2+^ ions may regulate cochlear physiology. Indeed, the impaired transfer of the Ca^2+^-mobilizing molecule inositol 1,4,5-trisphosphate [Ins (1,4,5)P_3_] has been suggested as a cause of recessive deafness due to a specific Cx26 mutation (Beltramello et al. 2005). The molecular selectivity of cochlear gap junctions has been explored extensively using dye transfer techniques. As in other tissues, these channels have the ability to select between molecules of different molecular weight and/or charge. Native cochlear gap junctions, in common with other Cx30-containing junctions (Eiberger et al. 2001; Forge et al. 2003a; Sun et al. 2005), often show a preference for positively charged species over negatively charged ones, and restrict the movement of molecules with a high molecular weight. In studies utilising a cochlear slice preparation (Forge et al. 2003a; Jagger and Forge 2006; Taylor et al. 2012), mature Deiters’ cells (supporting cells immediately adjacent to outer hair cells; Fig. 1b) allowed intercellular transfer of neurobiotin (molecular weight 287 Da, charge +1) but resisted the passage of Lucifer yellow (molecular weight 443 Da, charge −2). However, in Hensen’s cells (supporting cells at lateral border of organ of Corti), the passage of Lucifer yellow is less restricted. In the outer sulcus region of the adult guinea pig cochlea, Lucifer yellow transfers freely via gap junctions between root cells (Jagger et al. 2010).

The differential permeability to Lucifer yellow supports a varying expression of Cx26 and Cx30 in the organ of Corti, as demonstrated in Fig. 1c, and numerous other immuno-localization studies (Lautermann et al. 1998; Forge et al. 2003b; Sun et al. 2005). In various species, there is a higher expression of Cx30 in Deiters’ cells compared to Hensen’s cells (Sun et al. 2005; Jagger and Forge 2006; Zhao and Yu 2006; Liu et al. 2009; Forge et al. 2013). In addition, there are dramatic postnatal changes in the permeability properties of gap junctions in the organ of Corti. There is a rapid increase of dye-transfer between Deiters’ cells in rats during the first postnatal week, concomitant with increasing connexin immuno-reactivity (Jagger and Forge 2006). Though there is free transfer of Lucifer yellow between Deiters’ cells at P8, this likely reflects an immature phenotype, as this transfer is no longer apparent after the onset of hearing (>P12, see above). It is interesting to note that, contrary to the restricted transfer of Lucifer yellow between supporting cells of the organ of Corti, gap junctions between supporting cells in the avian inner ear are highly permeable for large anionic dyes (Nickel et al. 2006).

Although dye transfer experiments provide useful descriptions of gap junction characteristics, it is perhaps more pertinent to study the movement of endogenous messengers such as Ca^2+^ and inositol phosphates. These agents present a greater technical challenge as they themselves influence the physiology of the cells being studied. Gap junction-mediated Ca^2+^ waves have been monitored in HEK-293 cells expressing Cx26 and/or Cx30 (Sun et al. 2005). Ca^2+^ waves appear to spread significantly faster through groups of cells expressing heteromeric Cx26/Cx30 channels compared to groups of cells expressing homomeric Cx26 or homomeric Cx30 channels. Such differences have yet to be confirmed in functionally mature native cochlear tissue. In supporting cells of immature cochlear cultures, injection of Ins (1,4,5)P_3_ into a single cell initiated Ca^2+^ waves through neighbouring cells (Beltramello et al. 2005), suggesting an innate permeability of these gap junctions to this molecule. Of note is the observation that homomeric Cx26 channels in HeLa cells allow the passage of myo-inositol and various inositol polyphosphates, but heteromeric Cx26/Cx32 channels are much more selective, even distinguishing between isoforms of inositol triphosphate (Ayad et al. 2006). Comparable results have been observed for heteromeric Cx26/Cx30 channels (He et al. 2006). Gap junctions within the cochlear sensory epithelium of immature mice are permeable to fluorescent analogues of D-glucose (Chang et al. 2008), pointing to a role for connexins in the transport of energy substrates. Future studies must consider variations in connexin composition of channels throughout the mature cochlea, as these are all likely to have quite distinct properties. The distinct localisation of Cx30 to gap junctions between Deiters’ cells (Fig. 1c) suggests that larger anionic signals in particular will not easily transfer within that organisational compartment. Also, whilst much energy has been spent defining the physiology of the supporting cells in the organ of Corti, it is just as important to understand the contribution of the connective tissue gap junction network in the lateral wall to normal hearing (Kelly et al. 2011), and how its dysfunction leads to cochlear pathology.

## Do connexin hemichannels contribute to inner ear function?

A condition generally required for unpaired connexon channels to act as autonomous hemichannels is exposure to an extracellular medium largely devoid of divalent cations, particularly Ca^2+^ (Bennett et al. 2003). Whilst it is generally advisable to approach the area of hemichannels and their likely contribution to normal tissue homeostasis with some caution (Spray et al. 2006), the cochlea presents an unusual situation in which certain aspects of epithelial cells are exposed to an extracellular fluid which may theoretically permit hemichannel activation under normal circumstances. Endolymph within scala media is characterised as a fluid unusually rich in K^+^ (but low in Na^+^), but which also has an unusually low Ca^2+^ concentration, measured as approximately 20 μM in adult rats (Bosher and Warren 1978). The apical poles of some supporting cells are permanently exposed to the endolymph, thus raising the possibility of permanent activation of hemichannels. This has been adopted as the explanation for observed spontaneous ATP release from early postnatal cochlear cultures in vitro (Tritsch et al. 2007; Anselmi et al. 2008; Majumder et al. 2010; Schutz et al. 2010). Released ATP is proposed subsequently to activate purinergic receptors on the supporting cell luminal surface, which encourage the release of Ca^2+^ from intracellular stores in an IP_3_-dependent manner. Homomeric Cx26 gap junction channels in these immature tissues may then mediate intercellular transfer of Ca^2+^ and IP_3_ to instigate the regenerative propagation of intercellular Ca^2+^ waves, thus providing a system for long-distance signalling.

This complex interplay between gap junctions, hemichannels and purinergic receptors has been purported to play essential roles in the development of the cochlea (Dale 2008), and may explain observed spontaneous activity within the pre-hearing auditory nerve via the purinergic activation of immature hair cells (Tritsch et al. 2007; Johnson et al. 2011). This same mechanism has been proposed as a mediator of damage signals within the hearing cochlea (Gale et al. 2004). ATP has been measured at significant concentrations within the endolymph during normal hearing, and it rises during noise exposure (Munoz et al. 1995). Whether hemichannel-dependent purinergic signalling between supporting cells (and possibly hair cells) endures in vivo after hearing onset remains unproven, though ATP release has been reported from isolated adult cochlea, and this release was inhibited by raised Ca^2+^ or gap junction blockers (Zhao et al. 2005). Similarly, isolated adult supporting cells are reported to allow uptake of large anionic molecules in Ca^2+^-free conditions and this is reduced by physiological Ca^2+^ levels or applied gap junction blockers (Zhao 2005).

## Connexin mutations and deafness

The importance of gap junctional communication for auditory function has been highlighted by the discoveries that mutations in *GJB2* (coding for CX26), *GJB6* (CX30) and *GJB3* (CX31) may all cause hereditary hearing loss (Rabionet et al. 2002; Lee and White 2009; Xu and Nicholson 2013). Connexin mutations are associated with autosomal recessive and dominant hearing loss, whose phenotypes are mostly confined to the inner ear (non-syndromic) but can occur with other clinical features (syndromic), in particular skin disorders. Despite the genetic heterogeneity of non-syndromic autosomal recessive deafness (DFNB), a single locus on chromosome 13q11-12, DFNB1, accounts for up to 50 % of this type of hearing loss (Kenneson et al. 2002; Snoeckx et al. 2005). The gene responsible for DFNB1 has been identified as *GJB2* (Kelsell et al. 1997). Around 100, mostly recessive, mutations have been characterised in the *GJB2* gene, including splice, nonsense, missense and frame-shift mutations (see http://www.crg.es/deafness). Further complications may also arise following the identification of pathogenic mutations outside the coding region of *GJB2* (Matos et al. 2007). With a carrier frequency of 2–4 %, the most common mutation in European and North American populations is a deletion of a single guanine nucleotide, known as 35delG, which results in a frame-shift and the subsequent premature termination of protein translation. The majority of recessive Cx26 mutations studied to date do not form functional channels in recombinant expression systems, partly owing to impaired assembly of connexons, impaired targeting to the plasma membrane, or reduced protein stability (Martin et al. 1999; D’Andrea et al. 2002; Thonnissen et al. 2002; Oshima et al. 2003). However, several Cx26 mutations have been reported to form functional gap junction channels, albeit with reduced electrical coupling and impaired permeability for dye tracers (D’Andrea et al. 2002; Wang et al. 2003; Skerrett et al. 2004; Bicego et al. 2006). A subset of Cx26 mutant channels with amino acid substitutions at the second transmembrane domain (V84L, A88S and V95M) did not significantly affect electrical coupling, but impaired the transfer of larger molecules such as Ins (1,4,5)P_3_ (Beltramello et al. 2005; Zhang et al. 2005).

Several rare missense mutations in *GJB2* have been detected in families with autosomal dominant inheritance (DFNA3) (Denoyelle et al. 1998; Feldmann et al. 2005). These mutations primarily affect amino acids within the extracellular loops and result in impaired electrical coupling and dye transfer (Marziano et al. 2003; Chen et al. 2005; Piazza et al. 2005; Deng et al. 2006). In addition, dominant Cx26 mutations, in particular those that interfere with intracellular trafficking (Thomas et al. 2004), may be associated with various skin disorders (Richard et al. 1998, 2002; Maestrini et al. 1999; Heathcote et al. 2000; Uyguner et al. 2002). Currently, four deafness-causing recessive mutations at the DFNB1 locus have been reported. One encompasses the full DFNB1 locus, including both *GJB2* and *GJB6* genes (Feldmann et al. 2009). Two of them truncate the *GJB6* gene without affecting *GJB2* (del Castillo et al. 2002, 2005). Another deletion removes a 131-kb fragment in the DFNB1 region without affecting either *GJB2* or *GJB6* (Wilch et al. 2006), which supports the hypothesis that the deletions remove a regulatory element necessary for the expression of CX26 and/or CX30 in the inner ear. In fact, additional reports indicate that the expression of the *GJB2* allele in cis with either of the two deletions that truncate *GJB6* is dramatically reduced or switched off (Common et al. 2005; Rodriguez-Paris and Schrijver 2009). In addition, a missense mutation in *GJB6* affecting the amino-terminal of CX30 (T5M) is associated with non-syndromic autosomal dominant (DFNA3) middle to high-frequency hearing impairment with late onset (Grifa et al. 1999). Unlike CX30 mutations associated with skin disease, T5M mutants formed electrically coupled channels but showed impaired permeability for dye tracer and Ins (1,4,5)P_3_ (Common et al. 2003; Zhang et al. 2005; Schutz et al. 2010).

Mutations in the gene encoding connexin 31 (*GJB3*) have been detected in two different disorders: deafness (Xia et al. 1998; Liu et al. 2000) and erythrokeratodermia variabilis (Richard et al. 1998). Mutations linked to non-syndromic, dominant deafness (DFNA2) are concentrated at the second extracellular loop and do not form functional channels (He et al. 2005). The mild to moderate hearing loss is of late onset and, consistent with the Cx31 expression pattern (Xia et al. 2000), preferentially affects high frequencies (Xia et al. 1998). Autosomal recessive mutations located within the third transmembrane domain are associated with non-syndromic moderate to profound hearing loss (Liu et al. 2000).

## Insights and controversies arising from animal models

In agreement with the clinical phenotype of connexin mutations in humans, the deletion of Cx26 (Cohen-Salmon et al. 2002; Kudo et al. 2003) or Cx30 (Teubner et al. 2003) in the inner ear of mice results in severe hearing impairment (between 30–100 dB) shortly after the onset of hearing. The mechanism by which Cx30 deletion causes hearing impairment continues to be the subject of debate (see below). The ablation of Cx31 from the inner ear of mice does not result in morphological or functional defects (Plum et al. 2001). Differences between murine (Elfgang et al. 1995) and human Cx31 (Abrams et al. 2006) in their ability to form heteromeric/heterotypic gap junction channels may contribute to the disparities in the phenotype between mice and humans. In addition, while normal auditory function may not be compromised by the loss of Cx31, it would be interesting to study their susceptibility to noise and aging, especially as the absence of functional Cx31 channels is associated with progressive hearing loss. Conflicting reports have been presented for Cx29-deficient mice. While one study has found no effect of the deletion of Cx29 on auditory function (Eiberger et al. 2006), another using a different knockout mouse model detected demyelination of spiral ganglion neurons, which may be responsible for the prolonged latency and distortion in auditory brainstem responses, and higher sensitivity to noise damage observed in ∼50 % of these animals (Tang et al. 2006).

**Fig. 2:**
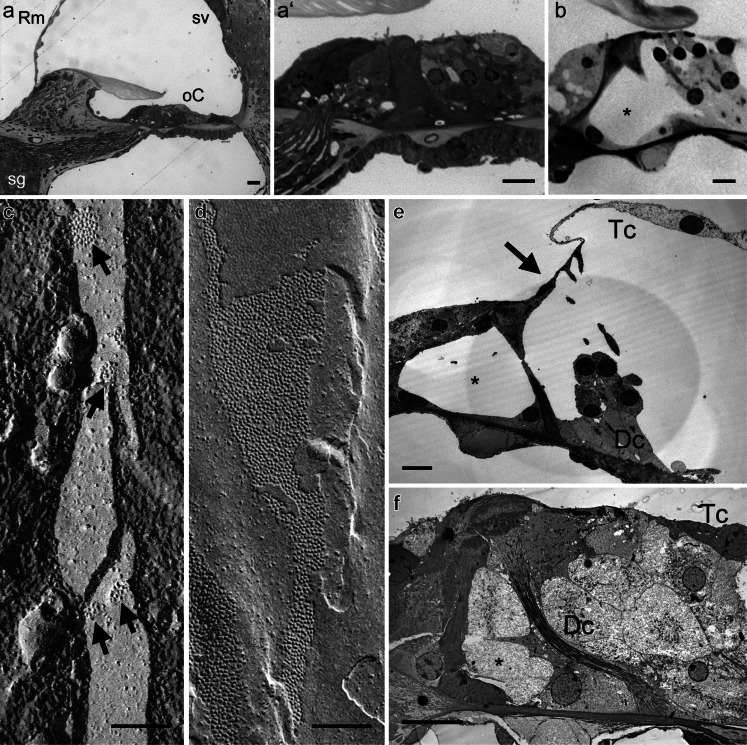
Cx26 and Cx30 play specific roles in development, membrane function and epithelial repair of the cochlea. **a**, **b** Light microscopy images of cochlear tissues. **a** In the basal turn of a 2-week old R75W-*Cx26* mouse, the essential sensory and non-sensory tissues are present, but on closer inspection the organ of Corti appears compact (**a’**). Deiters’ cells are short and intercellular spaces such as the tunnel of Corti and spaces of Nuel are absent. **b** In comparison, in a juvenile wild-type mouse, the organ of Corti has elongated Deiters’ cells and pillar cells, and a fully patent tunnel of Corti (*). **c**, **d** Freeze-fracture images of Deiters’ cell membranes in mice. Freeze-fracture reveals gap junction plaques as clusters of particles in the membrane. Each particle represents an individual channel (connexon). **c** In *Cx30-*null animals, plaques of the gap junctions between adjacent Deiters’ cells consist of only a few channels, and they are dispersed. **d** In wild-type animals, the plaques are extremely large (consisting of thousands of channels) and occupy a significant proportion of the membrane area. **e**, **f** Transmission electron micrographs of cochlear tissues. **e** Following loss of outer hair cells from a *Cx30*-null mouse, the Deiters’ cells remain columnar in shape, the tunnel of Corti is open (*), and the reticular lamina is displaced towards the pillar cells (*arrow*). **f** In a wild-type mouse treated systemically with kanamycin and bumetanide to cause loss of inner and outer hair cells, the Deiters’ cells have expanded to fill the gaps left behind by the hair cells. Cells with characteristics of Deiters’ cells have migrated between the outer pillar cells so that the tunnel of Corti appears filled (*). *Dc* Deiters’ cell, *oC* organ of Corti, *Rm* Reissner’s membrane, *sg* spiral ganglion, *sv* stria vascularis, *Tc* tectal cell. *Scale bars* (**a**, **b**, **e**, **f**) 10 μm, (**c**, **d**) 0.1 μm

In a mouse model for recessive Cx26-related deafness, the neonatal lethality of Cx26 knockout mice was overcome by restricting the deletion of Cx26 to the epithelial gap junction system (Cohen-Salmon et al. 2002). The inner ear of homozygous mice developed normally, but by postnatal day 14, soon after the hearing onset, apoptosis of supporting cells around the inner hair cells was observed, extending later to the outer hair cells and their supporting cells. It has been suggested that the death of supporting cells surrounding the inner hair cells is caused by oxidative stress owing to the interference of accumulated K^+^ with the removal of the neurotransmitter glutamate from the extracellular space (Cohen-Salmon et al. 2002). However, the specific properties of Cx26 that are required for hearing are called into question by the observation that its replacement by Cx32 results in no obvious cochlear deficits (Degen et al. 2011). In another mouse model, the function of Cx26 is inhibited by expressing the human R75W-*Cx26* mutation, which is associated with dominantly inherited hearing loss (Kudo et al. 2003). The dominant-negative effect of this missense mutation on the function of gap junction channels in vitro has been confirmed by electrophysiology and dye transfer approaches that demonstrated gap junctional communication was not only inhibited through homotypic R75W-Cx26 channels but also through heteromeric gap junction channels also containing wild-type Cx26 or Cx30 (Richard et al. 1998; Marziano et al. 2003). At 2 weeks of age, R75W-*Cx26* mutant mice displayed deformities of both the tunnel of Corti and the supporting cells (see Fig. 2a from our own studies of these mice) compared to those in wild-type mice (Fig. 2b). In the mutant mice, supporting cells fail to undergo essential maturation processes that occur just prior to the onset of hearing. These animals displayed profound increases of hearing thresholds (Kudo et al. 2003). By 7 weeks in R75W-*Cx26* mice, outer hair cells had degenerated, whereas inner hair cells were still present. No apparent effect was detected in the stria vascularis, which was confirmed by a normal endocochlear potential. These findings suggest that the pathogenicity of R75W-*Cx26* is confined to the epithelial gap junction system of the cochlea. The developmental defects observed in R75W-*Cx26* mutants were comparable to those in three mouse models with conditional deletions of Cx26 (Wang et al. 2009), further highlighting key roles that Cx26 plays in the differentiation of cells in the organ of Corti before the onset of hearing.

The genetic deletion of Cx30 from both the sensory epithelium and the connective tissues of the inner ear results in the failure to generate an endocochlear potential in 2-week old homozygous mutant (“*Cx30*-null”) mice despite apparently normal inner ear development and normal endolymphatic K^+^ concentrations (Teubner et al. 2003). From postnatal day 18 onwards, the apoptosis of hair cells was observed. In adult *Cx30*-null mice, the endolymphatic K^+^ concentration is also decreased, further contributing to the profound loss of hearing sensitivity. A subsequent study of these mutant mice reported that the key cellular components of the stria vascularis were all present and of apparently normal appearance (Cohen-Salmon et al. 2007). However, the unusual presence of serum proteins within stria vascularis, and marked abnormalities in the fine structure of strial capillaries, suggested the loss of endocochlear potential was caused by disruptions of the capillary endothelial barrier and a resulting intra-strial electrical shunt.

The question remains as to why both Cx26 and Cx30 are necessary for the maintenance of function in the cochlea. One explanation could be that the deletion of one connexin gene affects the expression of the other, resulting in an insufficient number of functional gap junctions in the cochlea. Although the immuno-cytochemical expression patterns of the remaining connexin are unchanged in mice with targeted deletions of Cx26 or Cx30 (Cohen-Salmon et al. 2002; Kudo et al. 2003; Teubner et al. 2003; Ahmad et al. 2007; Forge et al. 2013), Cx30 knock-out mice have Cx26 protein levels ∼25 % that of controls (measured by western blotting) despite unchanged Cx26 mRNA levels (Ahmad et al. 2007). A comparable decrease of Cx26 protein is also observed in a hearing-impaired knock-in mouse model of the *Cx30*-T5M mutation (Schutz et al. 2010). Together, these observations might suggest homotypic Cx26 channels are more unstable than heteromeric Cx26/Cx30 channels, which may lead to an insufficient number of functional gap junction channels. Indeed, in *Cx30*-null mice, the size and number of gap junction plaques between certain supporting cells is greatly reduced compared with those in normal animals (Fig. 2c, d). This reduced gap junction size between Deiters’ cells was reflected by decreased dye transfer between those cells in *Cx30*-null mice (Forge et al. 2013). However, between Hensen’s cells (where Cx26 is more highly expressed, Fig. 1d), plaque sizes were comparable between the genotypes, and dye transfer appeared unaffected. Lending further support to the hypothesis of reduced channel number is the observation that the over-expression of Cx26 restores hearing in Cx30-deficient mice (Ahmad et al. 2007).

It would seem, therefore, that Cx30 can be replaced by exogenous Cx26 to restore normal hearing, and that the biophysical properties of cochlear gap junctions are ultimately unimportant. Recently, a novel knock-out mouse was developed to further investigate the importance of Cx30 in normal auditory function. In the *Cx30* ^Δ/Δ^ model, Cx30 protein is completely absent and Cx26 protein is decreased, but to only ∼50 % of normal levels (Boulay et al. 2013). These mice hear normally, provoking a conclusion that Cx30 is dispensable for normal hearing. Further, the authors propose that dominant *GJB6* deafness mutations manifest themselves via their negative effects on heteromeric channels comprising normal Cx26 and mutated Cx30, and large deletions of *GJB6* affect *GJB2* expression. These observations in mice are consistent with those in humans where large deletions of *GJB3* may not result in hearing impairment (see above).

In work from our own laboratories employing the *Cx30*-null mouse model, we found that supporting cells were unable to carry out normal repair processes (Forge et al. 2013). Following hair cell loss in these mutant animals, the supporting cells fail to expand into the spaces left behind by the hair cells (Fig. 2e), and do not undertake the radial migration into the tunnel of Corti that occurs in normal animals pharmacologically induced to lose hair cells (Fig. 2f). Interestingly, during ototoxic hair cell loss in the chicken cochlea (“basilar papilla”), there is a decreased expression of Cx43 within supporting cells, a change associated with a loss of directional intercellular signalling (Jagger et al., in press, J Neuroscience). Furthermore, when remaining Cx30-containing gap junctions are blocked pharmacologically, normal repair processes are halted.

One remaining question that currently available mouse models have failed to answer is why specific human connexin deafness mutations result in hearing impairments that advance at different rates (Bitner-Glindzicz 2002). Some patients are profoundly deaf from birth (and presumably prenatally), yet others lose their hearing progressively over several years. A significant challenge to our field is to create biologically and clinically relevant models, which (1) can be used to attain a better understanding of the underlying pathologies occurring in human connexin deafness, and (2) can be exploited in order to develop effective ameliorative treatments to restore hearing.

## Conclusions

Building on the insights into functional properties of inner ear gap junctions and their mutations, often gained from studies in expression systems and immature cochlear cultures, the focus must now be turned to mature native tissues and their endogenous metabolites and second messengers. Increasingly refined animal models of connexin deletion will help us better recapitulate human inherited hearing loss within the laboratory setting. This will enable us to unravel the complexities of gap junctional communication in the inner ear and to better understand cochlear pathologies that result from connexin mutations. Great challenges lie ahead, not least delivering translational therapies within the clinical setting.
